# Real-world clinical outcomes of 5-aminolevulinic acid photodynamic therapy in patients with cervical intraepithelial neoplasia grade 1

**DOI:** 10.3389/fonc.2026.1896229

**Published:** 2026-10-21

**Authors:** Tianci Fan, Lina Geng, Guanwen Gao, Zixun Long, Degao Liu, Yunfei Wang, Changzhong Li

**Affiliations:** 1 School of Medicine, Southern University of Science and Technology, Shenzhen, Guangdong, China; 2 Department of Obstetrics and Gynecology, Peking University Shenzhen Hospital, Shenzhen, Guangdong, China; 3 Shenzhen Key Laboratory on Technology for Early Diagnosis of Major Gynecologic Diseases, Shenzhen, Guangdong, China; 4 Cheeloo College of Medicine, Shandong University, Jinan, Shandong, China; 5 Department of Obstetrics and Gynecology, Shenzhen Shenshan People’s Hospital, Shenzhen, Guangdong, China

**Keywords:** 5-aminolevulinic acid, cervical intraepithelial neoplasia grade 1, human papillomavirus, photodynamic therapy, ThinPrep cytologic test

## Abstract

**Background:**

Cervical intraepithelial neoplasia grade 1 (CIN1) is generally managed by observation because spontaneous regression is common and progression to high-grade disease is uncommon. Nevertheless, some patients with persistent HPV infection or repeated low-grade abnormalities may elect active treatment after appropriate counseling. This study aimed to describe the clinical outcomes and safety of 5-aminolevulinic acid photodynamic therapy (ALA-PDT) in patients with CIN1 who underwent active treatment.

**Methods:**

This retrospective cohort study included 269 patients with CIN1 who underwent ALA-PDT at Peking University Shenzhen Hospital between July 2021 and July 2025. All patients received six sessions of ALA-PDT at intervals of 7–14 days. HPV status and ThinPrep cytologic test (TCT) results were assessed before treatment and during follow-up at 3 months (± 1 month) and, in some patients, at 6 months (± 1 month) after treatment. HPV clearance, cytological improvement, and treatment-related adverse events were analyzed.

**Results:**

Among the 266 HPV-positive patients, 155 patients (58.27%) had a negative HPV result at the predefined 3-month follow-up. At 3 months, 205 of 247 patients (83.00%) with available cytological assessment had NILM cytology. No serious adverse events were observed during treatment or follow-up.

**Conclusions:**

In this retrospective cohort study, HPV negativity and improved cytological findings were observed during follow-up after ALA-PDT, together with an acceptable safety profile and no serious adverse events.

## Background

Cervical cancer remains one of the most common gynecological malignancies worldwide and continues to impose a substantial public health burden despite the widespread implementation of human papillomavirus (HPV) vaccination and cervical cancer screening programs. Although the incidence and mortality of cervical cancer have declined significantly in many countries, more than 300,000 women still die from cervical cancer annually worldwide (1). Persistent infection with high-risk HPV is recognized as the major etiological factor for cervical intraepithelial neoplasia (CIN) and cervical cancer development. Therefore, early identification and management of cervical precancerous lesions are essential for cervical cancer prevention (2). Therefore, most patients with CIN2+ undergo active treatment or close surveillance. In contrast, CIN1 is generally regarded as a low-grade manifestation of HPV-related cervical disease rather than a lesion that routinely requires treatment, and current guidelines recommend observation for most patients because of its high probability of spontaneous regression and low risk of progression (3, 4).

Compared with high-grade squamous intraepithelial lesions (HSIL/CIN2+), cervical intraepithelial neoplasia grade 1 (CIN1) is generally considered a low-grade lesion with a high spontaneous regression rate and a relatively low risk of progression to invasive cancer. Current guidelines therefore recommend observation rather than immediate treatment for most CIN1 patients (3). This recommendation is supported by evidence from large cohort studies and systematic review and meta-analytic evidence demonstrating that most CIN1 lesions regress spontaneously, whereas only a small proportion progress to CIN2+ (4). In a Norwegian cohort study involving 26,130 patients with CIN1, only 19% were diagnosed with CIN2+ within five years, and the cumulative incidence subsequently reached a plateau (5). Nevertheless, persistent HPV infection, repeated abnormal screening results, prolonged surveillance, and anxiety regarding disease progression may lead some patients to seek active treatment despite guideline-recommended observation.

Several non-excisional strategies have been investigated for patients with persistent HPV infection or low-grade cervical lesions, including topical immunomodulatory therapy, ablative techniques, therapeutic HPV vaccination, and PDT (6). However, the available evidence supporting these approaches remains limited, and no consensus has been reached regarding the optimal management strategy for patients who prefer treatment over observation.

Photodynamic therapy (PDT) is a minimally invasive treatment that selectively destroys abnormal epithelial cells through the interaction of a photosensitizer, light of a specific wavelength, and reactive oxygen species. PDT has been investigated in patients with persistent HPV infection and cervical intraepithelial lesions, including CIN1/LSIL. Randomized studies and systematic reviews have reported potentially favorable virological and lesion-related outcomes; however, the available evidence remains heterogeneous with respect to study populations, treatment protocols, comparators, and follow-up duration, and does not establish PDT as routine management for CIN1 (6–9). Unlike excisional procedures such as loop electrosurgical excision procedure (LEEP) and cervical conization, PDT does not require surgical removal of cervical tissue and may therefore be relevant to patients concerned about the potential future obstetric consequences of cervical excision (10, 11).

Although several studies have evaluated ALA-PDT in patients with HPV-related cervical lesions, evidence regarding its use in CIN1 remains limited and inconsistent, particularly in real-world clinical settings. Therefore, this retrospective study aimed to describe HPV negativity, cytological findings, and treatment tolerability in patients with CIN1 who underwent ALA-PDT in routine clinical practice, thereby providing additional real-world evidence to complement the existing literature.

## Methods

### Study design and patients

This single-center retrospective cohort study included patients diagnosed with cervical intraepithelial neoplasia grade 1 (CIN1) who underwent 5-aminolevulinic acid photodynamic therapy (ALA-PDT) at the outpatient clinic of Peking University Shenzhen Hospital between July 2021 and July 2025. Observation remained the guideline-preferred management strategy for CIN1. The patients included in this study represented a selected treatment-seeking population who underwent ALA-PDT after individualized clinical decision-making rather than a population for whom treatment was routinely indicated. Because the specific reasons for choosing ALA-PDT rather than observation were not systematically captured in the retrospective dataset, treatment selection could not be reliably categorized according to the duration of HPV persistence, persistence of CIN1, surveillance burden, patient preference, or other individual factors.

The study protocol was approved by the Ethics Committee of Peking University Shenzhen Hospital and conducted in accordance with the Declaration of Helsinki and relevant ethical guidelines for clinical research.

The inclusion criteria were as follows: (1) histologically confirmed CIN1 before ALA-PDT; (2) no prior cervical treatment, including CO_2_ laser therapy or excisional procedures, within 1 month before PDT; and (3) completion of follow-up evaluation at 3 months (± 1 month) after treatment, including at least HPV testing or ThinPrep cytologic test (TCT).

The exclusion criteria were as follows: (1) known allergy or hypersensitivity to photosensitizing agents or any excipients contained in the ALA preparation; (2) an interval exceeding 14 days between two consecutive treatment sessions, treatment discontinuation, or failure to complete all six scheduled PDT sessions; and (3) severe systemic diseases or clinically significant comorbidities that could interfere with treatment safety, outcome assessment, or follow-up compliance, including severe cardiovascular, hepatic, or renal dysfunction, autoimmune diseases, active infections, coagulation disorders, malignancies, psychiatric disorders, immunodeficiency conditions, or any other conditions considered unsuitable for ALA-PDT by the investigators.

### PDT protocol

All patients received ALA-PDT for a total of six treatment sessions at intervals of 7–14 days. Because no standardized treatment protocol for CIN1 currently exists, the treatment schedule was determined based on institutional clinical experience and previously reported PDT regimens for cervical intraepithelial lesions.

Before PDT, cervical mucus and vaginal secretions were gently removed using sterile cotton swabs, and the cervix was fully exposed using a vaginal speculum. A 20% solution of 5-aminolevulinic acid (ALA; 118 mg/vial; Fudan-Zhangjiang Bio-Pharmaceutical Co., Ltd., Shanghai, China) was prepared using sterile water, and 0.5 mL of the prepared solution was used for application. Cotton pads soaked with the prepared solution were applied to the cervical canal and ectocervix, extending approximately 0.5–1.0 cm beyond the visible lesion margins.

After an incubation period of 3 hours, the treatment area was irradiated using a photodynamic therapy device (Wuhan Yage Photoelectric Technology Co., Ltd., Wuhan, China) at a wavelength of 630 nm with an irradiance of 100 mW/cm^2^ for 25 minutes, corresponding to a total light dose of 150 J/cm^2^. During irradiation, the light field was carefully confined to the cervical treatment area whenever possible, and direct exposure of the vaginal wall was minimized. Although exposure of the vaginal wall to ALA was expected to be minimal during topical cervical application, these precautions were taken to avoid unnecessary photodynamic effects in adjacent normal tissues.

All procedures were performed and monitored by experienced gynecological specialists throughout the treatment course. During treatment, patients were instructed to avoid sexual intercourse and bathtub bathing.

### HPV testing and cytological evaluation

Cervical exfoliated cell specimens were collected for HPV testing and cytological evaluation before treatment and during follow-up. Qualitative detection of 21 HPV genotypes (6, 11, 16, 18, 26, 31, 33, 35, 39, 45, 51, 52, 53, 56, 58, 59, 66, 68, 73, 81, and 82) was performed using HPV nucleic acid assay kits manufactured by Sansure Biotech (Changsha, China). Cervical cytology was evaluated using the TCT, and cytological diagnoses were classified according to the 2014 Bethesda System (TBS).

### Outcome assessment

The primary endpoint of the study was HPV clearance at 3 months (± 1 month) after completion of ALA-PDT. Secondary endpoints included: (1) HPV status at the last available follow-up assessment; (2) exploratory descriptive analyses of subtype-specific HPV clearance, particularly for HPV16 and HPV18; (3) changes in TCT results before and after treatment; and (4) treatment-related adverse events. The 3-month (± 1 month) assessment was completed by all HPV-positive patients and therefore represented the fixed-time HPV outcome for the entire cohort. A subset of patients underwent an additional HPV assessment at 6 months (± 1 month); for these patients, the 6-month result was considered the last available HPV assessment. For patients with available follow-up data, HPV testing and TCT were additionally assessed at 6 months (± 1 month) after treatment completion. HPV clearance was defined as conversion from HPV-positive status before treatment to HPV-negative status during follow-up. Cytological improvement was defined as regression toward NILM status after treatment.

### Statistical analysis

All statistical analyses were performed using SPSS software version 26.0 (IBM Corp., Armonk, NY, USA). A two-sided P value <0.05 was considered statistically significant. Continuous variables were expressed as mean ± standard deviation (SD), whereas categorical variables were presented as frequencies and percentages. HPV clearance rates and TCT outcomes were summarized descriptively. The Sankey diagram illustrating changes in cytological outcomes before and after treatment was generated using the online visualization platform Flourish (https://flourish.studio/). Because follow-up duration and completeness varied across patients in this real-world retrospective cohort, and some patients discontinued surveillance after achieving reassuring clinical outcomes, paired statistical analyses were not consistently applicable. Therefore, the study primarily focused on descriptive analyses of virological and cytological outcomes during available follow-up.

## Results

### Patient characteristics

A total of 269 patients diagnosed with CIN1 were included in this retrospective cohort study. The mean age of the study population was 39.39 ± 9.41 years. The age distribution of the included patients is presented in **Figure 1**. Among all enrolled patients, 266 were HPV-positive at baseline, whereas 3 patients were HPV-negative. Among the HPV-negative patients, cytological examination showed atypical squamous cells of undetermined significance (ASC-US) in 1 patient and negative for intraepithelial lesion or malignancy (NILM) in 2 patients. Some HPV-negative patients were included because of persistent cytological abnormalities after clinical evaluation. Detailed baseline HPV infection status and TCT results are summarized in **Table 1**.

**Figure 1:**
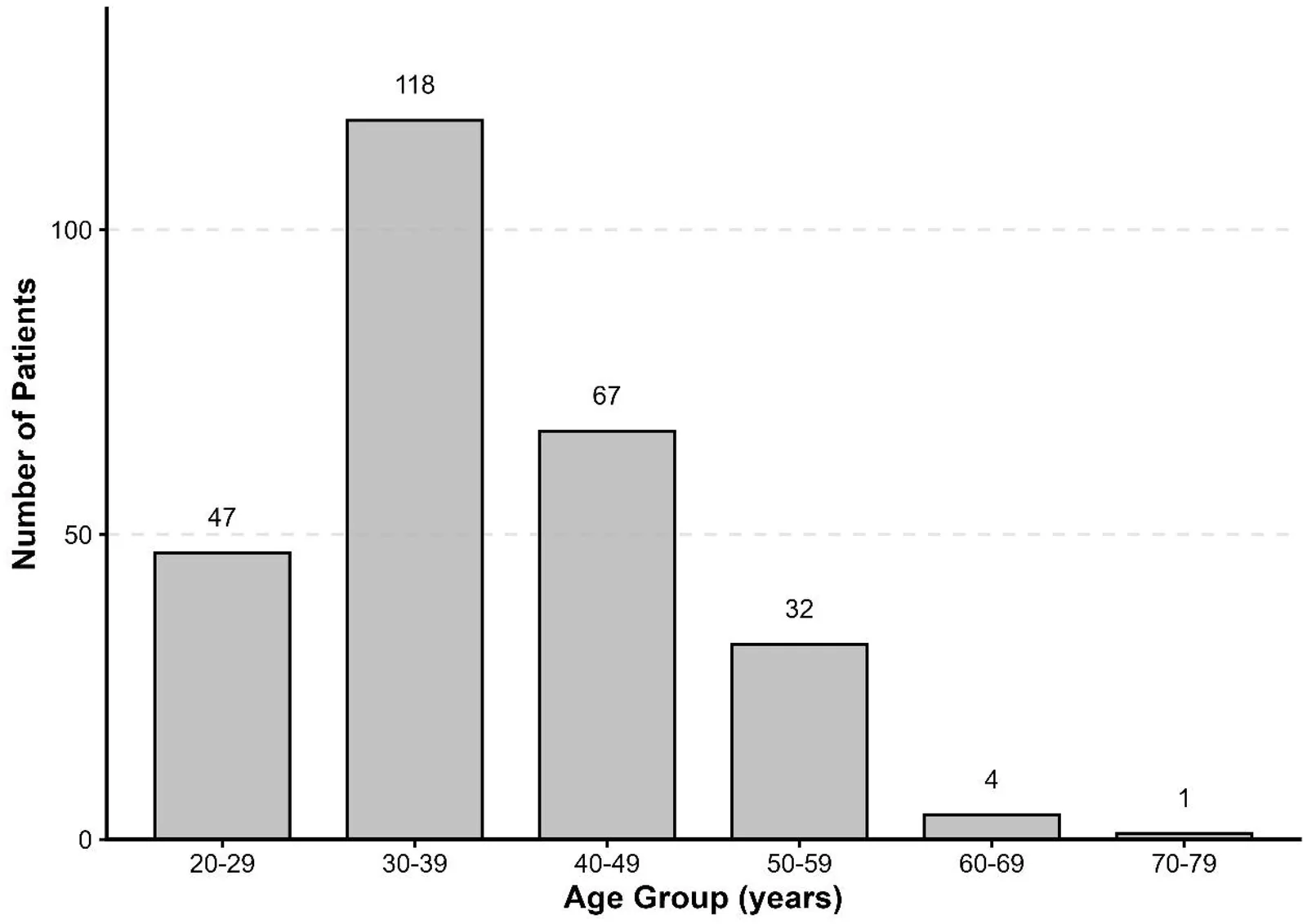
Age distribution of patients included in this study.

**Table 1:** Baseline characteristics of HPV infection status and TCT results.

Cytological findings	HPV16	HPV18	Other HPV
NILM	31	12	105
LSIL	9	2	49
ASC-US	11	7	57
ASC-H	0	0	2
AGC	0	0	1
Not checked	1	1	1
Total	52	22	215

The total number of HPV infections exceeds the number of enrolled patients because some patients had concurrent infections with multiple HPV genotypes.

### HPV outcomes

Among the 266 patients who were HPV-positive at baseline, all underwent the predefined HPV reassessment at 3 months (± 1 month) after completion of ALA-PDT, and 155 patients (58.27%) had a negative HPV result. A subset of patients underwent an additional HPV assessment at 6 months (± 1 month), whereas the remaining patients had no further HPV testing beyond the 3-month assessment. Therefore, the 3-month result represents the fixed-time HPV outcome for the entire HPV-positive cohort, whereas the last available HPV status combines the 3-month and 6-month assessments and is presented only as a descriptive supplementary outcome. Based on the last available HPV assessment, HPV negativity was documented in 170 of 266 patients (63.91%).

### Exploratory HPV16 subgroup analysis

A total of 52 patients were positive for HPV16 at baseline. At 3 months after treatment, HPV16 clearance was achieved in 34 of 52 patients (65.38%). Among the remaining HPV16-positive patients, 5 were lost to follow-up at 6 months. Among patients with available 6-month follow-up data, additional HPV-negative results were observed. Based on the last available HPV assessment, HPV negativity was documented in at least 39 of 52 patients.

### Exploratory HPV18 subgroup analysis

Twenty-two patients were positive for HPV18 before treatment. At 3 months after PDT, 21 patients achieved HPV clearance, corresponding to a clearance rate of 95.45%. The remaining patient did not complete further follow-up evaluation.

### Other HPV subtypes

A total of 215 patients were infected with HPV subtypes other than HPV16 or HPV18. At 3 months after treatment, 128 patients achieved HPV negativity, corresponding to a clearance rate of 59.53%. Among patients with persistent HPV positivity at 3 months, 39 underwent additional HPV testing at 6 months, and 19 subsequently converted to HPV-negative status. Based on the last available HPV assessment, HPV negativity was documented in at least 145 of 215 patients.

### Cytological outcomes

Among the 269 included patients, 266 underwent baseline TCT evaluation. Before treatment, 138 patients demonstrated NILM cytology, accounting for 51.88% of the study population. At 3 months after completion of ALA-PDT, 247 patients underwent repeat cytological evaluation, among whom 205 demonstrated NILM cytology, corresponding to 83.00%. Based on the last available cytological assessment, 227 patients had NILM cytology, accounting for 84.39% of the study population. Detailed changes in TCT results between baseline and the 3-month follow-up assessment are summarized in **Table 2**, and the dynamic transitions of cytological outcomes are illustrated in **Figure 2**. Cytological outcomes based on the last available follow-up assessment are provided in **Supplementary Table 1**.

**Table 2:** Comparison of cytological findings at baseline and 3 months after ALA-PDT.

Cytological findings	Baseline cytology (n=266)	3 months after ALA-PDT (n=247)
NILM	138(51.88%)	205(83.00%)
ASC-US	71(26.69%)	23(9.31%)
LSIL	54(20.30%)	19(7.69%)
ASC-H/HSIL/AGC	3(1.13%)	0(0.00%)

ASC-H, HSIL, and AGC were combined solely for descriptive presentation because each category contained very few cases. This grouping was intended to improve table readability and does not imply that these cytological categories are clinically equivalent.

**Figure 2:**
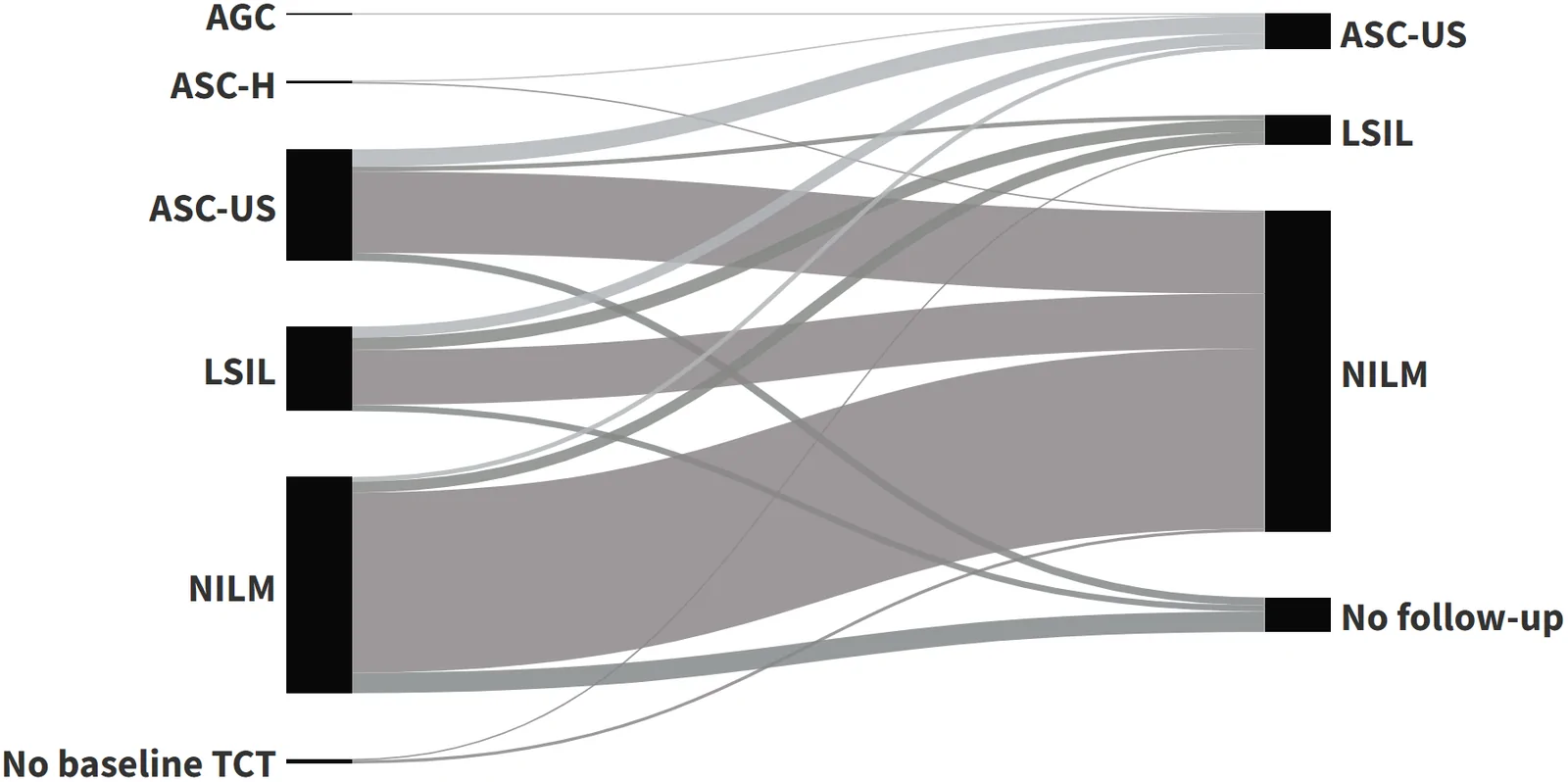
Sankey diagram showing dynamic transitions of TCT results between baseline and 3-month follow-up.

### Safety and adverse events

No serious adverse events were observed during treatment or follow-up. Some patients experienced mild treatment-related adverse reactions after the initial PDT session, including increased vaginal discharge, mild lower abdominal pain, and foul-smelling vaginal discharge. All symptoms improved after symptomatic treatment, including antibiotics or analgesics when necessary, and none of the adverse events resulted in treatment discontinuation or interruption of the treatment schedule.

## Discussion

In this retrospective cohort of patients with CIN1 who underwent ALA-PDT, HPV negativity and cytological improvement were observed during follow-up, with an acceptable safety profile and no serious adverse events. At the predefined 3-month assessment, 58.27% of baseline HPV-positive patients had a negative HPV result, and 83.00% of patients with available cytological assessment had NILM cytology. However, spontaneous HPV clearance and regression are common in CIN1; therefore, these observations should be interpreted cautiously and cannot be directly attributed to ALA-PDT in the absence of an untreated control group.

Current clinical guidelines generally recommend observation rather than immediate intervention for CIN1 because of its high spontaneous regression rate and relatively low risk of progression to invasive disease (2). Previous studies have shown that a substantial proportion of CIN1 lesions regress spontaneously without active treatment. Therefore, the improvements observed in the present study cannot be distinguished from the natural history of CIN1 (4). Nevertheless, persistent HPV infection, repeated surveillance, and uncertainty regarding disease progression may impose considerable psychological burden on some patients. In real-world clinical practice, a subset of women actively seek therapeutic intervention despite guideline-supported conservative management. Therefore, evaluating non-excisional approaches for carefully selected patients who elect active management after appropriate counseling remains clinically relevant.

Although observation remains the recommended management strategy for most patients with CIN1, some patients may elect active treatment after appropriate counseling, particularly in the setting of persistent HPV infection or repeated low-grade abnormalities. If ALA-PDT has a clinical role in CIN1, it should therefore be considered only for carefully selected treatment-seeking patients rather than for routine management. From a gynecologic oncology perspective, its potential relevance lies in evaluating a non-excisional approach within the cervical cancer prevention pathway while avoiding unnecessary cervical excision, rather than in treating CIN1 as a lesion that routinely requires oncologic intervention. Previous studies have reported that cervical excisional procedures may increase the risk of adverse obstetric outcomes, including preterm birth and premature rupture of membranes (10, 11). Therefore, PDT may be particularly relevant for selected patients who are concerned about the potential future obstetric consequences of cervical excisional treatment. However, fertility and pregnancy outcomes were not evaluated in the present study, and therefore no conclusions regarding these outcomes can be drawn.

HPV negativity was also observed during follow-up among patients infected with high-risk HPV subtypes such as HPV16 and HPV18. Although the exact mechanisms underlying HPV clearance after PDT remain incompletely understood, several biological mechanisms have been proposed. PDT induces the generation of reactive oxygen species, resulting in oxidative stress-mediated destruction of abnormal epithelial cells (12). In addition, accumulating evidence suggests that PDT may modulate local immune responses and enhance anti-HPV immunity (13, 14). Previous studies have demonstrated increased CD8+ T-cell infiltration and activation of tissue-resident memory T cells after PDT, which may contribute to viral clearance and long-term immune surveillance (14). Furthermore, Hisamatsu et al. reported increased anti-HPV16 antibody responses after ALA-PDT in patients with CIN2/3, suggesting that PDT may also stimulate HPV-specific humoral immunity. However, the specific contribution of HPV-specific immune responses, including antibody production, to HPV clearance after ALA-PDT remains unclear and requires further investigation (15).

Improvement in cytological findings was also observed during follow-up. The proportion of patients with NILM cytology increased markedly during follow-up, suggesting improvement of cervical cytological abnormalities in a considerable proportion of patients. Histopathological assessment remains the reference standard for evaluating CIN regression or progression; therefore, the observed cytological improvement should not be interpreted as evidence of histological regression because routine post-treatment histopathological reassessment was not performed. However, a small number of patients exhibited persistent or worsening cytological abnormalities after treatment. The reasons for persistent or worsening cytological abnormalities remain uncertain. Possible explanations include persistent HPV infection, transient inflammatory changes, or sampling variability during cytological evaluation; however, these hypotheses were not directly evaluated in the present study. Because this was a retrospective real-world study, histopathological reassessment was not routinely performed in patients who achieved both HPV negativity and NILM cytology during follow-up, which is consistent with routine clinical practice.

Safety remains an important consideration in PDT. In the present study, no serious adverse events were observed, and treatment-related symptoms were generally mild and transient. Most adverse reactions were limited to local discomfort, including increased vaginal discharge or mild lower abdominal pain, and improved with symptomatic management. Unlike systemic PDT used for other malignancies, topical ALA-PDT for cervical lesions results in minimal systemic photosensitizer exposure, thereby reducing the risk of systemic photosensitivity reactions.

This study has several strengths. First, to our knowledge, this is one of the relatively large real-world retrospective cohort studies evaluating ALA-PDT in patients with CIN1. Second, both virological and cytological outcomes were assessed, allowing a relatively comprehensive description of clinical outcomes following ALA-PDT. Third, exploratory subgroup analyses of HPV16 and HPV18 infections were performed, providing preliminary information regarding genotype-specific outcomes following ALA-PDT. Finally, all patients underwent a relatively standardized treatment protocol at a single institution, which improved treatment consistency.

Several limitations should also be acknowledged. First, this was a single-center retrospective study and therefore may be subject to selection bias and information bias. Second, some patients discontinued follow-up after achieving HPV negativity, resulting in incomplete long-term follow-up data. Third, HPV testing in this study was primarily qualitative rather than quantitative, limiting further analysis of viral load dynamics after PDT. In addition, histopathological follow-up data were unavailable for some patients because repeat cervical biopsy was not routinely performed in patients with reassuring HPV and cytological results during follow-up. More importantly, an untreated observational control group was not available; therefore, the observed changes in HPV status and cytology cannot be distinguished from spontaneous HPV clearance and regression expected during the natural history of CIN1. A light irradiation-only control group was also unavailable because this was a retrospective study of routine clinical practice, in which ALA administration and light irradiation were delivered together as the complete ALA-PDT intervention and light irradiation alone was not used as a clinical management strategy. Consequently, the specific contribution of ALA-mediated photodynamic effects could not be separately evaluated. In addition, because follow-up completion was influenced by clinical outcomes in some patients, selection bias associated with complete-case analysis could not be excluded. Therefore, formal paired statistical comparisons were not performed.

In this retrospective cohort of patients with CIN1 who underwent ALA-PDT, HPV negativity and cytological improvement were observed during follow-up, with acceptable tolerability. Because the study lacked an untreated observational control group, these findings cannot be attributed to ALA-PDT and should not be interpreted as evidence of treatment efficacy or as support for routine treatment of CIN1. Observation remains the recommended standard of care for most patients with CIN1. Prospective controlled studies using observation as the clinically relevant comparator, together with standardized follow-up and histopathological assessment, are needed to further define the potential role of ALA-PDT in selected patients.

## Data Availability

The raw data supporting the conclusions of this article will be made available by the authors, without undue reservation.
