# Supplementary material for: Real-world clinical outcomes of 5-aminolevulinic acid photodynamic therapy in patients with cervical intraepithelial neoplasia grade 1
Source: Front Oncol. 2026 Oct 21;16:1896229. doi: 10.3389/fonc.2026.1896229 (PMC13730827; doi:10.3389/fonc.2026.1896229)
Supplement: Supplementary file 1 [file DataSheet1.pdf]

Supplementary Table 1. Comparison of cervical cytological outcomes between baseline and final follow-up after ALA-PDT

Cytological findings	Baseline cytology (n=266)	Last available follow-up (n=269)
NILM	138(51.88%)	227(84.39%)
ASC-US	71(26.69%)	25(9.30%)
LSIL	54(20.30%)	17(6.32%)
ASC-H/HSIL/AGC	3(1.13%)	0(0.00%)
